# Hippocampal Long-Term Depression in the Presence of Calcium-Permeable AMPA Receptors

**DOI:** 10.3389/fnsyn.2018.00041

**Published:** 2018-11-13

**Authors:** Feng Cao, Zikai Zhou, Sammy Cai, Wei Xie, Zhengping Jia

**Affiliations:** ^1^Neurosciences & Mental Health, The Hospital for Sick Children, Toronto, ON, Canada; ^2^Department of Physiology, Faculty of Medicine, University of Toronto, Toronto, ON, Canada; ^3^The Key Laboratory of Developmental Genes and Human Disease, Southeast University, Nanjing, China; ^4^The Collaborative Innovation Center for Brain Science, Institute of Life Sciences, Southeast University, Nanjing, China

**Keywords:** GluA2, calcium-permeable AMPAR, hippocampal LTD, actin, cofilin, PICK1, NSF

## Abstract

The GluA2 subunit of AMPA glutamate receptors (AMPARs) has been shown to be critical for the expression of NMDA receptor (NMDAR)-dependent long-term depression (LTD). However, in young GluA2 knockout (KO) mice, this form of LTD can still be induced in the hippocampus, suggesting that LTD mechanisms may be modified in the presence of GluA2-lacking, Ca^2+^ permeable AMPARs. In this study, we examined LTD at the CA1 synapse in GluA2 KO mice by using several well-established inhibitory peptides known to block LTD in wild type (WT) rodents. We showed that while LTD in the KO mice is still blocked by the protein interacting with C kinase 1 (PICK1) peptide pepEVKI, it becomes insensitive to the N-ethylmaleimide-sensitive factor (NSF) peptide pep2m. In addition, the effects of actin and cofilin inhibitory peptides were also altered. These results indicate that in the absence of GluA2, LTD expression mechanisms are different from those in WT animals, suggesting that there are multiple molecular processes enabling LTD expression that are adaptable to physiological and genetic manipulations.

## Introduction

In the mammalian CNS, AMPA glutamate receptors (AMPARs) are the principal mediators of fast excitatory synaptic transmission and they are important in the expression of various forms of long-lasting synaptic plasticity, including long-term potentiation (LTP) and long-term depression (LTD; Malinow and Malenka, 2002; Bredt and Nicoll, 2003; Shepherd and Huganir, 2007; Collingridge et al., 2010; Huganir and Nicoll, 2013; Henley and Wilkinson, 2016). AMPARs are heteromeric complexes assembled from four distinct subunits, GluA1–4; however, in most principal neurons, AMPARs contain the GluA2 subunit (Wenthold et al., 1996; Isaac et al., 2007; Lu et al., 2009). The inclusion of GluA2 in the receptor complex confers a number of key properties of AMPARs, including Ca^2+^ permeability, receptor assembly and trafficking (Isaac et al., 2007). Accordingly, genetic and molecular ablation of GluA2 result in severe impairments in AMPAR assembly, synaptic physiology and behavior (Jia et al., 1996; Gerlai et al., 1998; Yan et al., 2002; Sans et al., 2003; Toyoda et al., 2007; Kessels and Malinow, 2009).

The study on the role of GluA2 in synaptic plasticity has been focused on its involvement in LTD expression (Malinow and Malenka, 2002; Collingridge et al., 2010). Ample previous studies have shown that the C-terminal domain (CTD) of GluA2 interacts with a number of synaptic proteins and that interference with these protein-protein interactions impairs AMPAR endocytosis and LTD expression (Nishimune et al., 1998; Osten et al., 1998; Song et al., 1998; Noel et al., 1999; Xia et al., 1999; Daw et al., 2000; Kim et al., 2001; Braithwaite et al., 2002; Lee et al., 2002; Seidenman et al., 2003; Jo et al., 2008; Terashima et al., 2008; Citri et al., 2010; Zhou et al., 2018). These results lead to a conclusion that GluA2 specifically controls LTD expression via AMPAR internalization mediated by its unique CTD-protein interactions (Malinow and Malenka, 2002; Collingridge et al., 2010). However, the results from genetic ablation experiments do not support this claim. In both GluA2 knockout (KO) and GluA2/3 double KO mice, NMDA receptor (NMDAR) dependent LTD can be induced (Jia et al., 1996; Meng et al., 2003; Asrar et al., 2009). In addition, in mice where all the AMPAR subunits (GluA1–3) were removed and replaced with kainate receptors, LTD was still expressed (Granger and Nicoll, 2014). These observations question the direct involvement of GluA2 in LTD expression.

There are a number of possibilities that may explain the presence of LTD in GluA2 and GluA2/3 KO mice. Most notably, complete absence of the GluA2 subunit, even with the cell/region specific KO approaches, may cause functional compensation in these mice. It is known that lack of GluA2, even if it is brief, can cause profound physiological and behavioral changes, including defects in receptor assembly, Ca^2+^ permeability and single channel conductance (Isaac et al., 2007; Liu and Zukin, 2007). For example, Ca^2+^ influx through GluA2 lacking, Ca^2+^ permeable AMPARs triggered by a brief high frequency stimulation, can induce synaptic plasticity (Jia et al., 1996; Asrar et al., 2009), which could in turn alter the homeostasis of synaptic physiology. Thus, synaptic plasticity in the GluA2 KO mice may utilize different mechanisms. Consistent with this possibility, more subtle changes in the CTD of GluA2, rather than the complete removal of the subunit, impairs LTD (Zhou et al., 2018).

In this study, we investigated hippocampal LTD at CA1 synapses in GluA2 KO mice by using a number of peptides and inhibitors known to affect LTD expression in wild type (WT) animals. We found that the effects of these manipulations are altered in GluA2 KO mice, indicating that mechanisms used in LTD expression in the absence of GluA2 are different from WT animals. Thus, there are multiple mechanisms that enable LTD expression at the CA1 synapses.

## Materials and Methods

### Mice, Peptides and Chemicals

Generation and genotyping of GluA2 KO mice were described previously (Jia et al., 1996). All the experimental procedures used for this research were conducted during the light cycle accordance with the guidelines of the animal care committees at the Hospital for Sick Children, Canada and Southeast University, China. The actin depolymerization inhibitor phalloidin was purchased from Thermo Fisher Scientific. Peptides S3 (MASGVAVSDGVIKVFN) that inhibits cofilin phosphorylation and pS3 (MASpGVAVSDGVIKVFN) that inhibits cofilin dephosphorylation were synthesized by GenScript and the APTC peptide synthesis facility. Active pep2m (KRMKVAKNAQ) that blocks GluA2-N-ethylmaleimide-sensitive factor (NSF) interaction and its control pep4c (KRMKVAKSAQ), active peptides pepEVKI (YNVYGIEEVKI) that blocks the GluA2-protein interacting with C kinase 1 (PICK1) interaction and the control pepSVKE (YNVYGIESVKE) were purchased from Tocris. Dynasore was purchased from Sigma Aldrich. The stock solutions for NMDA, DHPG, phalloidin and peptides (S3, pS3, 4c, 2m, SVKE, EVKI) were prepared in water, and for picrotoxin, Dynasore and MPEP in DMSO. The stock solutions were diluted 2,000× with artificial cerebrospinal fluid (ACSF) for field recordings or 500× with the intracellular solution for whole-cell recordings right before use.

### Slice Electrophysiology

The detailed slice recording procedures and analysis in Schaffer/Collateral pathway in the hippocampus were described previously (Meng et al., 2002, 2003; Zhou et al., 2011). The mouse brains were quickly removed and sagittal 350 μm hippocampal slices were prepared in ice-cold ACSF saturated with 95% O_2_/5% CO_2_. ACSF contained (in mM): 120 NaCl, 3.0 KCl, 1.2 MgSO_4_, 1.0 NaH_2_PO_4_, 26 NaHCO_3_, 2.0 CaCl_2_ and 11 D-glucose. Slices were recovered at 22–26°C for at least 2 h before recording. A single slice was transferred to a submersion chamber perfused with 95% O_2_/5% CO_2_ saturated ACSF with (for whole-cell recordings) or without (for field recordings) 100 μM picrotoxin. Perfusion flow rate was set at 2 ml per min. Hippocampal CA1 neurons were visualized using an infrared differential interference contrast microscope (Olympus X51 or Zeiss Axioscope). Synaptic transmission was evoked at 0.05 Hz for field recordings and 0.1 Hz for whole-cell recordings of the Schaffer/Collaterals pathway onto the CA1 pyramidal neurons and recorded with glass pipettes (3–4 MΩ) filled with either ACSF (for field recordings) or the intracellular solution (for whole-cell recordings) containing (in mM) 130 CsMeSO_4_, 5 NaCl, 1 MgCl_2_, 0.05 EGTA, 10 HEPES, 3 Mg-ATP, 0.3 Na_3_GTP and 5 QX-314 (pH 7.25; 280–300 mOsm). For whole-cell experiments, cells were clamped at −65 mV throughout the experiments. Whole-cell series resistance was monitored by applying a −3 mV step at the end of each response sweep. The experiment was excluded from analysis if resistance changed by more than 20%. For peptide infusion experiments, the lack of the effect of each peptide on basal synaptic responses was tested independently by recording baseline in the presence of the peptides for at least 1 h without LTD induction. Young (2–3 weeks) and mature (2–3 months) WT and GluA2 KO littermates were used in this study. Experimenters were blind to the genotype of the mice. Field EPSP (fEPSP) LTD induction protocols used in this study were: low-frequency stimulation (LFS, 900 pulses at 1 Hz), paired-pulse LFS (PP-LFS, 900 pairs of pulses at 1 Hz with 50 ms pairing interval), NMDA bath perfusion (25 μM for 3 min) or DHPG bath perfusion (100 μM for 10 min). LTD of whole-cell EPSC recordings was induced by LFS (300 pulses at 1 Hz) delivered at −30 mV holding potential or PP-LFS (600 pairs of pulses at 1 Hz with 50 ms pairing interval) or 100 μM DHPG perfusion for 10 min. The mGluR antagonist MPEP (40 μM) was perfused 10 min before and 5 min after PP-LFS stimulation. All data acquisition and analysis were done using pClamp 10.6 software (Axon Instruments, Sunnyvale, CA, USA). In all electrophysiological experiment figures, *n* represents the number of neurons or slices and at most two slices per animal were used. All the experiments were performed with KO and their aged matched WT littermate control. The data were statistically evaluated with Student’s *t*-test. The error bars in all figures represent SEM. The average data of the last 20 min (40–60 min after LTD induction) of field recordings and the last 10 min (30–40 min after LTD induction) of whole-cell recordings were used for *t*-test comparison.

### Slice Treatment and Western Blot Analyses

Hippocampal slices used for treatment and western blot analyses were prepared and maintained as for electrophysiological recordings. The detailed procedures for western blot analyses were described previously (Huang et al., 2011). Acutely prepared hippocampal slices were recovered for at least 2 h at 22–26°C in 95% O_2_/5% CO_2_ saturated ACSF, and then transferred to a treatment chamber perfused with 95% O_2_/5% CO_2_ saturated ACSF for additional 30 min recovery before DHPG treatment. The hippocampal slices treatment experiments were divided into two groups: slices removed immediately before DHPG application were defined as untreated control (DHPG 0 min) and slices treated with 100 μM DHPG for 10 min as DHPG treated group (DHPG 10 min). Hippocampal slices were frozen in dry ice immediately and stored at −20°C at the end of each treatment. Samples were lysed for 40–50 min in ice-cold lysis buffer containing (in mM) 20 Tris pH 7.5, 150 NaCl, 1 EDTA, 1 EGTA, 1% Triton X-100, 2.5 sodium pyrophosphate, 1 β-glycerophosphate, 1 Na_3_VO_4_, 20 NaF, 1 μg/ml leupeptin, 1 PMSF and 0.5% protease inhibitor cocktail (Calbiochem) and phosphatase inhibitor (Roche). The supernatant was collected by centrifugation at 12,000 rpm (4°C) for 10 min. The total protein concentration of each sample was measured via BCA assay. Ten microgram of total protein samples of each group were loaded on SDS gel. Proteins were separated on 12% SDS-PAGE polyacrylamide gel and electrotransferred to a nitrocellulose filter. Filters were then blocked with 2% dry milk in TBST (20 mM Tris base, 9% NaCl, 0.1% Tween-20, pH 7.6) and incubated overnight at 4°C with cofilin (Cell Signalling Technology) or p-cofilin (Cell Signalling Technology) antibodies in TBST. Following washing and incubating with secondary antibodies, membranes subjected to chemiluminescence signal detection using a Pierce HRP kit. Protein band intensity was analyzed using the AlphaEaseFC software. GAPDH (Santa Cruz Biotechnology) was used as protein loading control. *N* in the experiments represents the number of independent experiments.

## Results

### Age-Dependence for the Requirement of GluA2 in Hippocampal LTD

**Figure 1:**
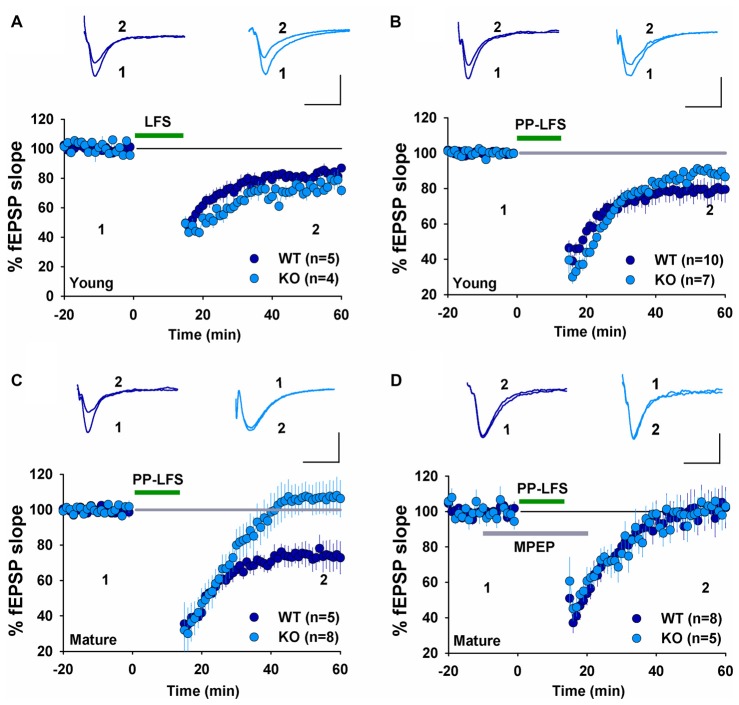
Field EPSP (fEPSP) recordings showing long-term depression (LTD) expression in young, but not mature GluA2 knockout (KO) mice. **(A)** Low-frequency stimulation (LFS) induces LTD in both young wild type (WT) and GluA2 KO mice. **(B)** Paired-pulse LFS (PP-LFS) induces LTD in both young WT and GluA2 KO mice. **(C)** PP-LFS induces LTD in mature WT but not in mature GluA2 KO mice. **(D)** MPEP (40 μM, bath perfusion 10 min before, during and 15 min after the PP-LFS stimulation) blocks PP-LFS induced LTD in mature WT, but has no effect on the absence of PP-LFS induced LTD in GluA2 KO mice. Scale bars: 0.5 mV/10 ms for field recordings and 50 pA/50 ms for whole-cell recordings.

**Figure 2:**
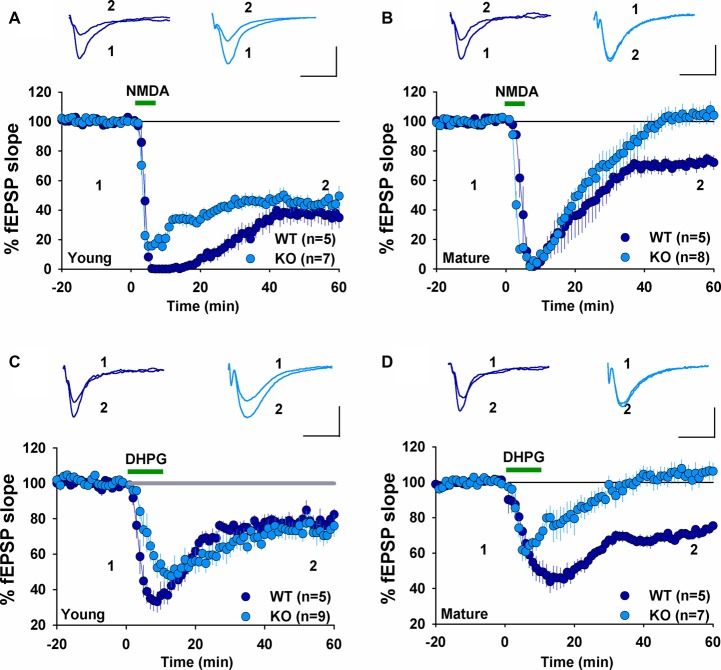
fEPSP recordings showing chemically induced LTD in young, but not mature GluA2 KO mice. **(A)** NMDA (25 μM, 3 min) induces LTD in both young WT and GluA2 KO mice. **(B)** NMDA (25 μM, 3 min) induces LTD in mature WT but not in mature GluA2 KO mice. **(C)** DHPG (100 μM, 10 min) induces LTD in both young WT and GluA2 KO mice. **(D)** DHPG (100 μM, 10 min) induces LTD in mature WT but not in mature GluA2 KO mice.

We have previously shown that NMDAR-dependent LTD induced by LFS (900 pulses at 1 Hz) was normal in 2–3 week old GluA2 KO and GluA2/3 double KO mice (Jia et al., 1996; Meng et al., 2003). In contrast, mGluR-LTD induced by DHPG was impaired in 6–7 week old GluA2 KO mice (Zhou et al., 2011). These results suggest that the requirement for GluA2 might be specific to either the induction trigger (NMDA or mGluR) or developmental stage. To distinguish these possibilities, we analyzed NMDAR- and mGluR-LTD in both young (2–3 weeks) and mature (2–3 months) mice. First, we confirmed our previous field potential recording results in young mice. LFS (900 pulses at 1 Hz), which is known to induce NMDAR-LTD, elicited a similar amount of LTD in both WT and GluA2 KO mice (Figure 1A, WT: 82.2 ± 2.5%, *n* = 5; KO: 74.9 ± 4.5%; *n* = 4; *p* > 0.05). Since LTD is difficult to induce in slices obtained from adult tissue, we used PP-LFS (900 paired pulses at 1 Hz with a 50 ms inter-pulse interval) that readily induces LTD in both young and mature rodents. As shown in Figures 1B,C, although PP-LFS induced a similar small LTD in both WT and KO mice at the young age (Figure 1B, WT: 78.8 ± 5.9%, *n* = 10; KO: 89.6 ± 4.4%, *n* = 7; *p* > 0.05) and a robust LTD in mature WT mice (Figure 1C, WT: 74.1 ± 6.7%, *n* = 5), LTD was absent in mature KO mice (Figure 1C, KO: 106.1 ± 9.1%, *n* = 8; *p* < 0.05 compared with WT). To test whether the PP-LFS induced LTD was mGluR-dependent, we bath applied the mGluR antagonist MPEP (40 μM) 10 min before, during and 5 min after PP-LFS stimulation. The LTD was blocked in WT mice (Figure 1D, WT: 97.5 ± 8.7%, *n* = 8; *p* > 0.05 compared to baseline) and remained absent in mature KO mice (Figure 1D, KO: 98.6 ± 6.1%, *n* = 5; *p* > 0.05 compared to baseline), suggesting that PP-LFS induced LTD in mature WT mice requires mGluRs. These results indicate that the requirement for GluA2 is age-dependent. To further test this idea, we pharmacologically induced LTD by addition of specific agonists for NMDARs or mGluRs. To test for NMDAR-LTD, we used a brief (3 min) bath application of 25 μM NMDA. As shown in Figures 2A,B, although this form of LTD was obtained in both young WT and KO mice (Figure 2A, WT: 37.5 ± 6.5%; *n* = 5; KO: 46.3 ± 4.7%, *n* = 7; *p* > 0.05) and mature WT mice (Figure 2B, WT: 71.0 ± 4.2%, *n* = 5), it was absent in mature KO mice (Figure 2B, KO: 102.6 ± 4.4%, *n* = 8; *p* < 0.05). To test for mGluR-LTD, we used bath application of 100 μM group I mGluR agonist DHPG (10 min; Palmer et al., 1997). As shown in Figures 2C,D, although this form of LTD was induced in both young WT and KO mice (Figure 2C, WT: 78.2 ± 3.9%, *n* = 5; KO: 73.5 ± 5.2%, *n* = 9; *p* > 0.05) and mature WT mice (Figure 2D, WT: 70.7 ± 1.9%, *n* = 5), it was absent in mature KO mice (Figure 2D, KO: 104.2 ± 5.2%, *n* = 7; *p* < 0.05). Taken together, these results indicate that GluA2 is required for both NMDAR- and mGluR-LTD in mature, but not in young mice.

### Effect of Actin and Cofilin Inhibitors in Young GluA2 KO Mice

**Figure 3:**
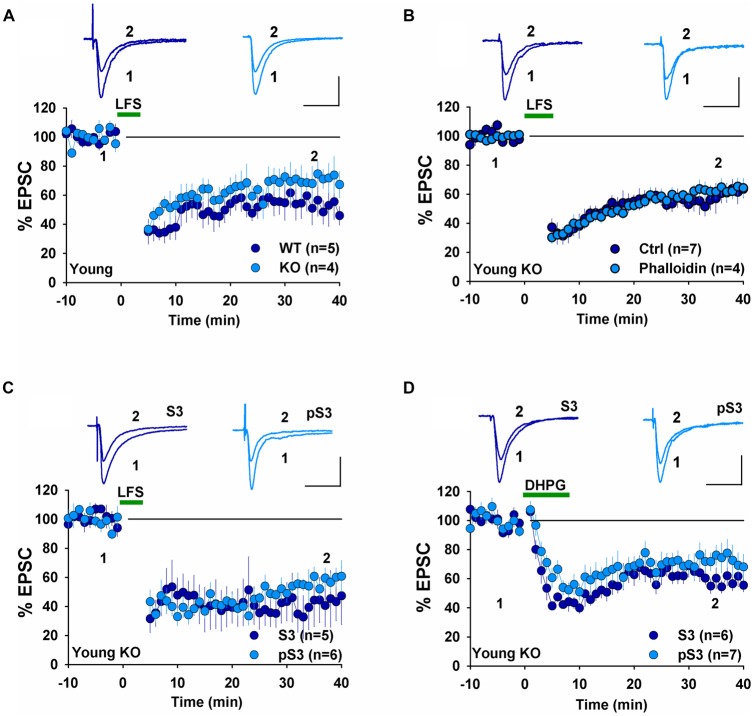
Effect of phalloidin and cofilin peptides on NMDA receptor (NMDAR)-LTD in young GluA2 KO mice. **(A)** LFS induces LTD in both young WT and GluA2 KO mice in whole-cell recordings. **(B)** Phalloidin has no effect on LFS-induced LTD in young GluA2 KO mice. **(C)** Cofilin inhibitory pS3 or enhancing peptide S3 has no effect on LFS-induced LTD in young GluA2 KO mice. **(D)** pS3 or S3 has no effect on DHPG-induced LTD in young GluA2 KO mice.

The presence of NMDAR-LTD in young GluA2 KO appears to be at odds with many previous studies showing that GluA2 is required for this form of LTD. However, it is possible that the germline deletion of the entire GluA2 subunit may have altered synaptic physiology enabled LTD expression in the KO mice. Therefore, for the rest of the study, we focused on LTD mechanisms in GluA2 KO mice. First, we tested the effects of perturbing the actin cytoskeleton. Actin filaments are the predominant cytoskeleton element in dendritic spines and their dynamic changes are important for spine morphology and synaptic plasticity (Cingolani and Goda, 2008). Our previous studies demonstrated that cofilin-mediated actin depolymerization is required for LTD expression (Zhou et al., 2011) and this requirement appears to be age-dependent (Cao et al., 2017). We first confirmed that under whole-cell recording mode without any inhibitor infusion, LFS induced an indistinguishable amount of LTD in WT and GluA2 KO mice (Figure 3A, WT: 53.9 ± 10.3%, *n* = 5; KO = 68.5 ± 9.4, *n* = 4, *p* > 0.05). To determine the role of actin depolymerization, we performed the same experiments in GluA2 KO mice but included the actin depolymerization inhibitor phalloidin in the recording electrode. As shown in Figure 3B, infusion of 100 μM phalloidin had no effect on LTD in young GluA2 KO mice (Figure 3B, Ctrl = 58.0 ± 7.3%, *n* = 7; phalloidin = 62.4 ± 5.3%, *n* = 4; *p* > 0.05). Cofilin is a potent regulator of actin reorganization in response to neuronal activity and is known to be critically involved in spine regulation (Bamburg, 1999; Meng et al., 2002; Huang et al., 2011). To determine the involvement of cofilin, we tested two peptides, S3 and pS3 known to increase and decrease the activity of cofilin respectively (Aizawa et al., 2001; Huang et al., 2011) in GluA2 KO mice. As shown in Figure 3C, neither S3 nor pS3 peptides had an effect on LTD (Figure 3C, S3: 42.2 ± 13.0%, *n* = 5; pS3: 55.6 ± 5.5%, *n* = 6; *p* > 0.05) in young GluA2 KO mice. Similar results were seen in DHPG-induced LTD, where no effect was found either S3 or pS3 in young GluA2 KO mice (Figure 3D, S3: 59.4 ± 5.3%, *n* = 6; pS3: 72.3 ± 9.3%, *n* = 7; *p* > 0.05). These results show that, similar to WT animals (Cao et al., 2017), cofilin-mediated actin reorganization is not required for both LFS- and DHPG-induced LTD in young GluA2 KO mice.

### Altered Effects of NSF Inhibitory Peptides in Young GluA2 KO Mice

**Figure 4:**
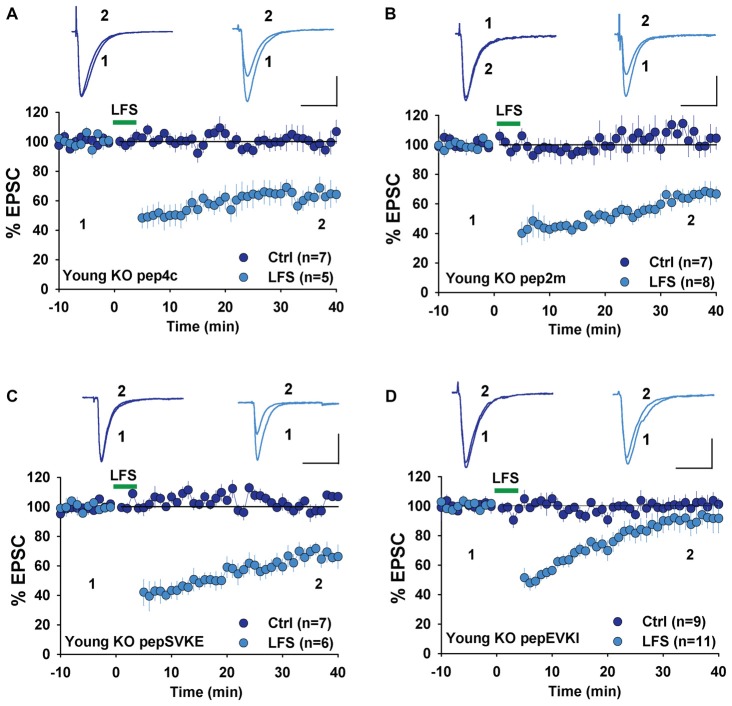
Effect of N-ethylmaleimide-sensitive factor (NSF) and protein interacting with C kinase 1 (PICK1) peptides on NMDAR-LTD in young GluA2 KO mice. **(A)** pep4c has no effect on either basal synaptic responses or LFS-induced LTD in young GluA2 KO mice. **(B)** pep2m has no effect on either basal synaptic responses or LFS-induced LTD in young GluA2 KO mice. **(C)** pepSVKE has no effect on either basal synaptic responses or LFS-induced LTD in young GluA2 KO mice. **(D)** pepEVKI inhibits LFS-induced LTD without affecting basal synaptic responses in young GluA2 KO mice.

Next, we examined the involvement of GluA2 interacting proteins, specifically NSF and PICK1, both of which are known to be required for LTD in WT rodents. We first used a peptide, pep2m that disrupts the interaction between GluA2 and NSF. This peptide has been shown to causes a run-down of AMPAR-mediated basal synaptic transmission (Nishimune et al., 1998; Song et al., 1998) and to prevent LTD in rats (Lüscher et al., 1999; Lüthi et al., 1999; Lee et al., 2002). We have previously shown that pep2m impairs both basal synaptic transmission and LTD expression in WT mice (Cao et al., 2017). Therefore, we tested the effect of pep2m in young GluA2 KO mice. As shown in Figure 4A, the control peptide pep4c had no effect on either basal synaptic transmission (Figure 4A, Ctrl: 100.8 ± 3.2%, *n* = 7; *p* > 0.05 compared to responses at the beginning of the recording) or LTD (Figure 4A, LFS: 63.6 ± 2.2%, *n* = 5; *p* > 0.05 compared to LTD without any peptide in Figure 3A) in young GluA2 KO mice. The active peptide pep2m also had no effect on basal synaptic transmission (Figure 4B, Ctrl: 105.3 ± 3.9%, *n* = 7; *p* > 0.05 compared to the responses at the beginning of the recording) or LTD expression (Figure 4B, LFS: 65.2 ± 3.5%, *n* = 8, *p* > 0.05 compared to LTD with pep4c). These results are in clear contrast with those in WT animals, suggesting that LTD in young GluA2 KO mice is no longer NSF-dependent.

### Effects of PICK1 Inhibitory Peptides in Young GluA2 KO Mice

In WT animals, pepEVKI, a peptide that prevents the interaction between GluA2 and PICK1, inhibits LTD expression (Daw et al., 2000; Kim et al., 2001; Seidenman et al., 2003; Jo et al., 2008; Terashima et al., 2008; Citri et al., 2010) although it does not invariably do so (Daw et al., 2000). These results suggest that GluA2-PICK1 interaction is required for LTD. However, because PICK1 can interact with many other proteins (Xu and Xia, 2007; Hanley, 2008), whether its role in LTD regulation is indeed mediated by GluA2 is unclear. To address this question, we compared the effects of pepEVKI and a control peptide, pepSVKE (Daw et al., 2000) on the baseline response and LFS-induced LTD in young GluA2 KO mice. We found that while the control peptide, pepSVKE, had no effect on either basal synaptic transmission (Figure 4C, Ctrl: 101.5 ± 3.7%, *n* = 7; *p* > 0.05 compared to the responses at the beginning of the recording) or LTD (Figure 4C, LFS: 66.3 ± 4.8%, *n* = 6; *p* > 0.05 compared to LTD without any peptide), the active peptide pepEVKI, while having no effect on basal synaptic transmission (Figure 4D, Ctrl: 100.8 ± 4.6%, *n* = 9; *p* > 0.05 compared to the responses at the beginning of the recording), significantly diminished LTD in young GluA2 KO mice (Figure 4D, LFS: 90.9 ± 4.9, *n* = 11; *p* < 0.05 compared to LTD with pepSVKE in Figure 4C). These results suggest that although PICK1 is still required for LTD expression in young GluA2 KO mice, this requirement is no longer GluA2 dependent.

### LTD Mechanisms in Adult GluA2 KO Mice

**Figure 5:**
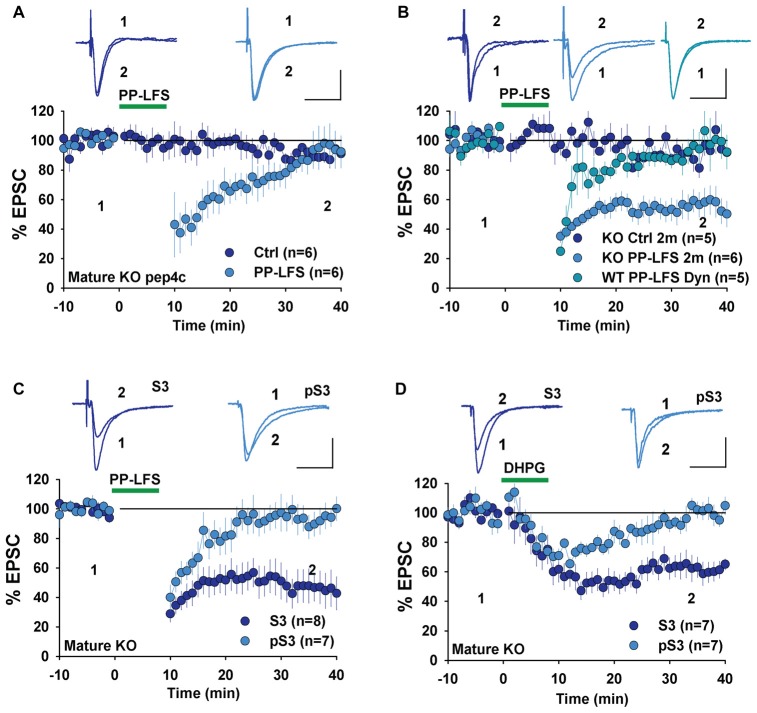
mGluR-LTD in mature GluA2 KO mice. **(A)** pep4c has no effect on either basal synaptic responses or PP-LFS induced LTD in mature GluA2 KO mice. **(B)** pep2m has no effect on basal synaptic responses, but rescues LTD induced by PP-LFS in mature GluA2 KO mice. Dynasore (80 μM) blocks PP-LFS induced LTD in WT mice. **(C)** S3 but not pS3 rescues PP-LFS induced LTD in mature GluA2 KO mice. **(D)** S3 but not pS3 rescues DHPG-induced LTD in mature GluA2 KO mice.

To investigate whether changes in LTD mechanisms in young GluA2 KO mice were also found in mature mice, we examined LTD in adult GluA2 KO mice. Since LTD is not easily induced by LFS in mature rodents, we used the PP-LFS protocol that typically induces an mGluR-dependent form of LTD (Kemp et al., 2000; Figure 1D). As shown in Figure 5A, pep4c had no effect on basal synaptic transmission and LTD was absent (Figure 5A, Ctrl: 88.2 ± 3.2%, *n* = 6; PP-LFS: 90.4 ± 9.0%, *n* = 6; *p* > 0.05) in mature GluA2 KO mice. The active peptide, pep2m, also had no effect on baseline synaptic transmission (Figure 5B, Ctrl: 93.5 ± 5.9%, *n* = 5; *p* > 0.05 compared to the responses at the beginning of the recording). Interestingly, pep2m restored LTD in adult GluA2 KO mice (Figure 5B, PP-LFS: 52.5 ± 8.3%, *n* = 6; *p* < 0.05). To test whether the PP-LFS induced LTD in mature mice was endocytosis dependent, we applied the endocytosis inhibitor Dynasore (80 μM) and found that LTD was blocked (Figure 5B, WT: 95.6 ± 10.1%, *n* = 5; *p* > 0.05 compared to baseline). These results are in clear contrast to mature WT animals where pep2m blocks LTD expression (Cao et al., 2017), suggesting that the requirement for NSF in adult GluA2 KO mice is also altered. We previously showed that the cofilin inhibitory peptide pS3 blocks LTD whereas the activation peptide S3 has no effects in mature WT mice (Cao et al., 2017). Therefore, we also tested the effects of these peptides in mature GluA2 KO mice. As shown in Figure 5C, while pS3 had no effect, S3 afforded a full rescue of LTD in the KO mice (Figure 5C, S3: 53.0 ± 10.9%, *n* = 8; pS3: 92.9 ± 8.2%, *n* = 7; *p* < 0.05). Similar results were obtained for DHPG-induced LTD where S3, but not pS3, rescued LTD expression in adult GluA2 KO mice (Figure 5D, S3: 62.3 ± 2.7%, *n* = 7; pS3: 98.4 ± 3.5%, *n* = 7; *p* < 0.05). These results suggest that the status of cofilin mediated actin organization is altered in adult GluA2 KO mice.

### Age-Dependent Changes in Cofilin in GluA2 KO Mice

**Figure 6:**
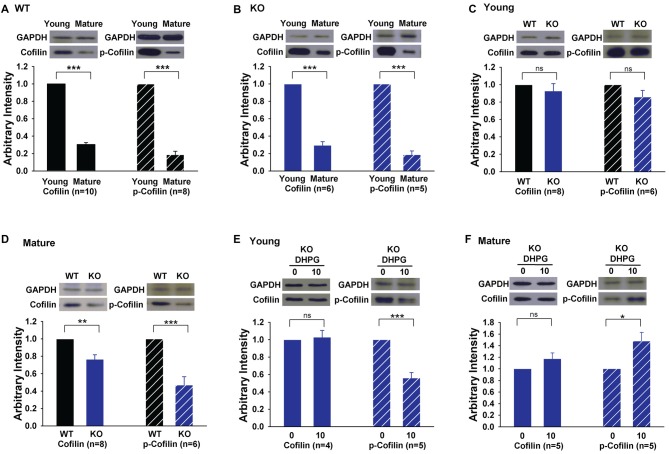
Expression of cofilin and phosphorylated cofilin (p-cofilin) in young and mature GluA2 KO mice. **(A,B)** Western blots showing that both total baseline cofilin and p-cofilin decrease in mature compared to young mice in both WT and GluA2 KO mice. ***p < 0.001 compared with young mice. **(C,D)** Western blots showing that baseline cofilin and p-cofilin are similar in WT and GluA2 KO young mice but significantly reduced in mature GluA2 KO mice compared to mature WT mice. **p < 0.01, ***p < 0.001 compared with WT mice, ns: not significant (*p* > 0.05). **(E,F)** DHPG activates cofilin in young but not in mature GluA2 KO mice. *p < 0.05, ***p < 0.001 compared with untreated group, ns: not significant (*p* > 0.05).

The results that the activation of cofilin by S3 rescued LTD in GluA2 KO mice suggest that the level and/or activity of cofilin may be altered in the KO mice. To test this possibility, we analyzed total and phosphorylated (inactive) cofilin. First, we found that both the total protein level and activity of cofilin were significantly lower in mature compared to young WT mice (Figure 6A, cofilin: 0.30 ± 0.02, *n* = 10, *p* < 0.001; p-cofilin: 0.19 ± 0.04, *n* = 8, *p* < 0.001). This developmental down regulation of cofilin was also seen in GluA2 KO mice (Figure 6B, cofilin: 0.30 ± 0.04, *n* = 6, *p* < 0.001; p-cofilin: 0.19 ± 0.04, *n* = 5, *p* < 0.001). In young mice, there was no difference in either total or phosphorylated cofilin between WT and GluA2 KO mice (Figure 6C, cofilin, 0.93 ± 0.08, *n* = 8, *p* > 0.05; p-cofilin, 0.86 ± 0.07, *n* = 6, *p* > 0.05). However, in mature mice, both total and phosphorylated cofilin were reduced in Glu2A KO mice (Figure 6D, cofilin, 0.77 ± 0.05, *n* = 8, *p* < 0.01; p-cofilin, 0.47 ± 0.09, *n* = 6, *p* < 0.001). To investigate activity-dependent cofilin changes in GluA2 KO mice, we examined the effect of DHPG treatment on cofilin. As shown in Figures 6E,F, the amount of total cofilin was not affected by DHPG in both young and mature GluA2 KO mice (Figure 6E, Young: 1.03 ± 0.08, *n* = 4, *p* > 0.05 compared with untreated; Figure 6F: Mature: 1.17 ± 0.1, *n* = 5, *p* > 0.05 compared with untreated). However, the level of p-cofilin was decreased after DHPG treatment in young GluA2 KO mice (Figure 6E, 0.56 ± 0.06, *n* = 5, *p* < 0.001 compared with untreated). Interestingly, unlike the young GluA2 KO mice, the amount of p-cofilin was significantly increased after DHPG treatment in mature GluA2 KO mice (Figure 6F, 1.48 ± 0.15, *n* = 5, *p* < 0.05 compared with untreated), which suggests that DHPG failed to activate cofilin in mature GluA2 KO slices (Figure 6F). These data are consistent with the idea that LTD in mature slices requires cofilin activation and this process is altered in GluA2 KO mice.

## Discussion

There are a number of conclusions from the present study. First, GluA2 appears to be differentially required at different developmental stages. While GluA2 is dispensable in slices obtained from young mice, it is required for LTD in slices obtained from mature mice. Second, in young slices, although LTD can be induced, the underlying mechanisms are different, being NSF-independent and PICK1-dependent. Third, although LTD is impaired in mature GluA2 KO mice, it can be restored by manipulating NSF or cofilin, suggesting that the basic machinery for LTD expression is still present in these KO mice.

The finding that NMDAR-LTD was indistinguishable in slices from WT and KO young mice indicate that NMDAR-LTD can be induced in the absence of GluA2 at this age. The presence of NMDAR-LTD in GluA2 KO mice appears to be in conflict with the previous studies based on the peptide studies (Lüscher et al., 1999; Lüthi et al., 1999; Lee et al., 2002) but can be explained if we propose that the KO mice use different mechanisms for NMDAR-LTD expression. Previous studies showed that pep2m reduces basal synapse response and occludes NMDAR-LTD (Lüscher et al., 1999; Lüthi et al., 1999). But in GluA2 KO mice, the peptide exerts no effects on either basal synaptic response or NMDAR-LTD, suggesting that NSF is no longer involved in NMDAR-LTD expression in the KO mice. It is possible that GluA1 is sufficient for NMDAR-LTD expression if GluA2 is absent. It is possible that the dephosphorylation of Ser845 of GluA1 (Lee et al., 2000, 2003) may play a dominant role in NMDAR-LTD in GluA2 KO mice. Alternatively, in KO mice there may be an AMPAR independent endocytosis mechanism in operation that mediates the LTD (Granger and Nicoll, 2014). In contrast to pep2m, pepEVKI was able to inhibit NMDAR-LTD in GluA2 KO similar to the WT mice. This peptide has been shown to prevent the binding of PICK1, but not GRIP, to the C-terminal tail of GluA2, and thus its ability to block LTD has been attributed to disruption of PICK1 binding to GluA2 interaction. However, the present finding that pepEVKI was just as effective in the KO argues against this notion. Rather it is consistent with disruption of an action of PICK1 downstream of GluA2, as suggested by previous studies (Citri et al., 2010). It has been shown that during NMDAR-LTD, PICK1 is activated by Arf1 and inhibits the Arp2/3 complex (Rocca et al., 2008, 2013; Nakamura et al., 2011). Arp2/3 is a key protein complex required for actin reorganization (Pollard, 2007). It is possible that, by binding to PICK1, pepEVKI impairs the ability of PICK1 to regulate the Arp2/3-mediated formation of filament networks, resulting in NMDAR-LTD inhibition.

In adult tissue, mGluR-LTD induced by either using PP-LFS or DHPG appears to require GluA2 because both forms of mGluR-LTD are absent in GluA2 KO mice. However, we found that either pep2m or S3 is able to fully restore LTD in the KO mice. This suggests that the mGluR-LTD process is fully present in the KO but that it is acutely inhibited in some way as a result of the absence of GluA2. One possibility is that whereas in WT mice synaptic NSF is mainly bound to GluA2, in KO mice it is freely available to bind other substrates and in doing so it impairs mGluR-LTD. Pep2m then prevents this NSF-mediated inhibition of LTD. One potential substrate of NSF is PICK1. It is known that NSF forms a protein complex with GluA2 and PICK1 and that synaptic recruitment of AMPARs by NSF requires disruption of the GluA2-PICK1 interaction (Hanley et al., 2002). It is possible that, in the GluA2 KO, free NSF binds the PICK1 protein complex in a manner that impairs mGluR-LTD, and that pep2m prevents this interaction. This is consistent with the observation that in GluA2 KO mice, the pepEVKI peptide still blocks mGluR-LTD. It is important to note that the peptide pep2m is not specific to NSF, but also inhibits AP-2 protein interactions (Lee et al., 2002). In fact, it was shown that more NSF-specific peptides (e.g., pep-R845A that binds NSF, but not AP-2) cause a run-down of AMPAR-mediated currents without an effect on LTD. Therefore, it is possible the effect of pep2m on LTD in GluA2 KO mice could be mediated by AP-2 rather than NSF. Clearly more experiments are necessary to distinguish these possibilities.

The requirement for actin reorganization in mature mice is also disrupted in GluA2 KO mice. In WT mice, mGluR-LTD is prevented by the pS3 peptide that blocks cofilin activation (Zhou et al., 2011; Cao et al., 2017), suggesting cofilin-mediated actin depolymerization is necessary. In adult GluA2 KO mice, mGluR-LTD is impaired but was fully rescued by the S3 peptide that increases cofilin activation, which is shown to have no effects on mGluR-LTD in WT mice. These data argue that mGluR-LTD mechanisms involving cofilin and actin are altered in GluA2 KO mice. This argument is consistent with the biochemical data that the absence of GluA2 resulted in significantly less cofilin in mature tissue. In addition, stimulation of mGluRs is associated with dephosphorylation (activation) of cofilin in WT but not in KO mice. The absence of cofilin activation in adult KO mice may explain the loss of mGluR-LTD. In young tissue, mGluR stimulation is able to activate cofilin, irrespective of the presence of GluA2.

In summary, we demonstrate that in GluA2 KO mice, the mechanisms involved in both NMDAR- and mGluR-LTD are significantly different from those of WT animals. Therefore, there are multiple mechanisms that enable LTD expression at CA1 synapses, and it would be important to dissect the details of these mechanisms and their contributions to overall plasticity and behavior.

## Author Contributions

ZJ conceived the study. ZJ, FC and ZZ designed the experiments. FC, ZZ and SC performed the experiments. FC and SC analyzed the data. ZJ and FC wrote the article. WX made suggestions to the manuscript. All authors read and approved the manuscript.

## Conflict of Interest Statement

The authors declare that the research was conducted in the absence of any commercial or financial relationships that could be construed as a potential conflict of interest.

## Abbreviations

AMPARs: AMPA receptors
CTD: c-terminal domain
fEPSP: field EPSP
KO: knock out
LFS: low-frequency stimulation
LTD: long-term depression
LTP: long-term potentiation
NMDARs: NMDA receptors
NSF: N-ethylmaleimide-sensitive factor
PICK1: protein interacting with C kinase 1
PP-LFS: paired-pulse low-frequency stimulation
WT: wild type.
